# TPM-like manipulations exerted an antidepressant effect and regulated amino acid metabolism and GHR/IGF-1 pathway in the liver in adolescent female CUMS rats

**DOI:** 10.3389/fphys.2026.1752608

**Published:** 2026-05-14

**Authors:** Shaoyun Zhao, Jiaxuan Gong, Rong Wu, Ying Xiong, Yun Gu, Yuhang Wang, Zhixiu Song

**Affiliations:** 1 College of Acupuncture Moxibustion and Tuina, Nanjing University of Chinese Medicine, Nanjing, Jiangsu, China; 2 Department of Traditional Chinese Medicine, Qinghai University Medical College, Xining, China; 3 Key Laboratory of Acupuncture and Medicine Research of Ministry of Education, Nanjing University of Chinese Medicine, Nanjing, Jiangsu, China; 4 College of Health and Rehabilitation, Nanjing University of Chinese Medicine, Nanjing, Jiangsu, China

**Keywords:** amino acid metabolism, depression, IGF-1, liver-brain axis, massage

## Abstract

**Objective:**

To explore whether traditional-pediatric-massage-like (TPM-like) manipulations exert antidepressant effects and regulate amino acid metabolism and GHR/IGF-1 pathway in the liver from the liver-brain axis perspective in adolescent female CUMS rats.

**Methods:**

Female Sprague–Dawley rats (3–4 weeks) were randomly divided into four groups (n=7–8): control (CON), chronic unpredictable mild stress (CUMS), CUMS + TPM-like manipulations (TPM), and CUMS + fluoxetine (FLX). Depressive-like behaviors were assessed using the sucrose preference test, open field test, and Morris water maze test. Hippocampal insulin-like growth factor 1 (IGF-1) and IGF-1 receptor (IGF-1R) expressions were detected by qRT-PCR and WB. Hippocampal and hepatic tumor necrosis factor alpha (TNF-α) and interleukin-1β (IL-1β) were assessed by qRT-PCR, and hepatic histopathology was evaluated by HE staining. Hepatic growth hormone receptor (GHR) and IGF-1 expressions were detected by qRT-PCR and WB. Liver metabolism was assessed by UPLC-MS/MS. Hepatic L-Aspartic Acid and plasma IGF-1, alanine aminotransferase (ALT), and aspartate aminotransferase (AST) were measured by ELISA. Associations among liver metabolites, L-Aspartic Acid, depressive-like behaviors, and GHR/IGF-1 expression were analyzed.

**Results:**

CUMS induced depressive-like behaviors, inflammation and reduced expressions of IGF-1 and IGF-1R in the hippocampus in adolescent female rats. Moreover, CUMS significantly disturbed amino acid metabolism, particularly lowered the concentration of L-Aspartic Acid, reduced the expressions of GHR/IGF-1 pathway in the liver, induced liver inflammation. TPM-like manipulations exerted antidepressant effects, maintained amino acid metabolic homeostasis in the liver and mitigated inflammation in both the hippocampus and the liver. Importantly, the concentration level of L-Aspartic Acid was negatively related to the severity of depressive-like behaviors, positively related to the expression levels of IGF-1 and IGF-1R in the hippocampus as well as GHR and IGF-1 in the liver.

**Conclusion:**

TPM-like manipulations prevented CUMS-induced depressive-like behaviors, increased concentration of L-Aspartic Acid, increased the expressions of GHR and IGF-1 in the liver, increased the expressions of IGF-1 and IGF-1R, and inhibited inflammation in the hippocampus in adolescent female rats. However, further study is warranted to draw confirmative results related to liver-brain axis.

## 1 Introduction

Adolescent depression is a pressing global public health challenge, with a rising prevalence and a significant gender disparity emerging after puberty, with a much higher risk for females than males (Salk et al., 2017). Depression severely impairs social function and elevates suicide risk in adolescents (Lewinsohn et al., 2001; Thapar et al., 2022). Hippocampal neuroinflammation is considered an important contributor to adolescent depression, which may be driven by microglial activation and cytokine overproduction under chronic stress (Zhang et al., 2022b; Liu et al., 2024; Wu et al., 2024; Zhang et al., 2025). Insulin-like growth factor 1 (IGF-1), a potent neurotrophic factor, has an important role in neuroprotective and anti-inflammatory functions (Arroba et al., 2016). IGF-1 is abundantly expressed throughout the central nervous system, of which the production peaks during the critical developmental period of adolescence (Yamamoto et al., 1991; Loomba-Albrecht and Styne, 2009). Circulating IGF-1 is a major source of IGF-1 in the central nervous system, including the hippocampus (Trejo et al., 2001; Pharaoh et al., 2020).

The liver is the major source of circulating IGF-1, contributing approximately 75% to the systemic pool in a process primarily driven by pituitary-derived GH (Puche and Castilla-Cortázar, 2012; Staerk et al., 2020). The binding of GH to growth hormone receptor (GHR) on the hepatocyte membrane triggers the gene transcription of IGF-1 in the liver (Fan et al., 2009; Hu et al., 2021). Therefore, the liver is a major upstream regulator of circulating IGF-1. An increasing number of studies indicate that chronic stress induces pathological changes, inflammation, and metabolic disturbances in the liver, which may impair hepatic IGF-1 production (Jia et al., 2016; Maiers and Chakraborty, 2023). Several studies showed that liver damage was accompanied by alterations in IGF-1 expression (Stanley et al., 2021; Schneider et al., 2025).

An increasing number of studies proves that the liver-brain axis plays an important role in the occurrence and development of depression. The liver may influence the brain through metabolic, inflammatory, and endocrine signals, including alterations in amino acid metabolism and liver-derived factors (Sun et al., 2024; Yang et al., 2024; Song et al., 2025). Accumulating evidence suggests that disturbed liver metabolism is associated with depression- and anxiety-related symptoms, as well as cognitive impairment (Shea et al., 2024; Meroni et al., 2025). CUMS can induce liver inflammation and liver dysfunction (Xu et al., 2022; Pang et al., 2023), which further contributes to depression. Furthermore, L-Aspartic Acid plays a significant role in alleviating liver inflammation and fibrosis. It exerts this effect by inhibiting the activation of inflammasomes and reducing the expression of pro-inflammatory cytokines, thus alleviating acute liver injury and preserving liver tissue integrity (Zhou et al., 2023). Additionally, long-term exposure to aspartic acid has been shown to enhance the expression of N-methyl-D-aspartic acid receptor subunits in the hippocampus, which contributes to the preservation of intermediate-term spatial memory and supports synaptic plasticity in the hippocampal region (Zachar et al., 2021).

As a non-pharmacological therapeutic approach, massage has been reported to alleviate symptoms of depression and anxiety with relatively few adverse effects (Moyer et al., 2004). In traditional Chinese medicine (TCM), back stroking and spine-pinching manipulations are core components of traditional pediatric massage (Lin et al., 2024; Liu et al., 2025). In TCM, spine-pinching is a classical manipulation applied along the dorsal midline and adjacent paraspinal region, corresponding topographically to the Du vessel and the Foot-Taiyang Bladder meridian (Lin et al., 2022; Tao et al., 2025). Traditionally, these regions are considered important for regulating visceral functions (Lin et al., 2022; 2024). From a modern perspective, however, the effects of this manipulation are more cautiously interpreted as patterned mechanical stimulation of cutaneous and paraspinal tissues that may engage peripheral sensory afferents, spinal-autonomic pathways, and higher-order central networks involved in affective and visceral regulation (Zhao, 2008; Bai et al., 2009; Zhang et al., 2020; Lin et al., 2024). In the present preclinical study, the intervention was operationalized as a standardized traditional-pediatric-massage-like (TPM-like) manipulations designed to model the core biomechanical features of the back-based clinical procedure, rather than to reproduce the full human pediatric practice. Previous work showed that chronic unpredictable mild stress (CUMS) induced depressive-like behaviors, hippocampal inflammatory changes, reduced hippocampal IGF-1 expression, and slower body weight gain in early adolescent rats (Wu et al., 2024; Zhang et al., 2025). Previous studies from our team showed that back stroking alone produced weaker effects than TPM-like manipulations, suggesting that gentle tactile stimulation alone was insufficient to account for the full response (Wu et al., 2024). Based on this prior evidence and in accordance with the principles of Replacement, Reduction, and Refinement, an additional sham control group was not introduced in the current experiment. Under these conditions, TPM-like manipulations attenuated depressive-like behaviors, reduced hippocampal inflammatory changes, increased hippocampal IGF-1 expression, and improved body weight gain (Zhang et al., 2025). Because IGF-1 is a major downstream effector of the GH/IGF-1 axis and an important regulator of somatic growth (Heemskerk et al., 1999), these findings raise the possibility that stress-related reductions in hippocampal IGF-1 may be associated, at least in part, with altered hepatic IGF-1 production and circulating IGF-1 availability.

In TCM theory, the liver has long been considered closely related to emotional regulation, a concept that may broadly parallel the emerging liver–brain axis framework in modern biomedicine (Chen et al., 2020; Yan et al., 2023). Based on this background, the present study was designed to explore whether TPM-like manipulations exert antidepressant-like effects from the perspective of the liver–brain axis, with particular focus on hepatic metabolism, hepatic GHR/IGF-1-related signaling, and their associations with behavioral and hippocampal changes in early adolescent female rats exposed to CUMS.

## 2 Materials and methods

### 2.1 Animals

Female Sprague-Dawley rats (3–4 weeks old at study initiation; Slaccas Experimental Animal Co. Ltd., Shanghai, China), corresponding to the early adolescent period (Spear, 2000), were housed four per cage under standard conditions (22 ± 2 °C, 30%–40% humidity, 12-h light/dark cycle) with ad libitum access to food and water. All rats were allowed to acclimatize to the housing conditions for 3 days before randomization and the initiation of the experimental procedures. All animal procedures were approved by the Animal Care and Use Committee of Nanjing University of Chinese Medicine (No. 202405A074).

### 2.2 Experimental design

**Figure 1:**
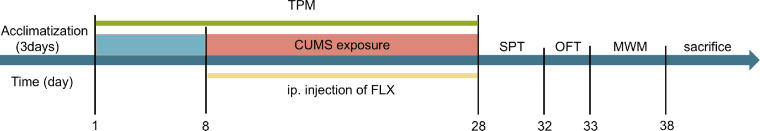
Experiment protocol. CON, control; CUMS, chronic unpredictable mild stress; TPM, traditional pediatric massage-like manipulations; FLX, fluoxetine.

After 3 days of acclimatization, rats were randomly assigned to four groups (n = 7–8 per group): control (CON), CUMS, CUMS + traditional-pediatric-massage-like manipulations (TPM), and CUMS + fluoxetine (FLX). The experimental design is shown in (**Figure 1**). CUMS exposure and group-specific interventions were conducted over a 28-day period (days 1–28). Following the completion of CUMS modeling and interventions on day 28, behavioral testing was performed sequentially: the sucrose preference test (SPT) began on day 28, the open field test (OFT) was conducted on day 32, and the Morris water maze (MWM) was performed on days 33–38. All animals were sacrificed after completion of the final MWM probe trial for tissue and blood collection.

### 2.3 CUMS exposure

Except for the CON group, all rats were individually housed in a separate experimental room under the same standard conditions. The CUMS procedure is a widely validated and effective method for inducing depression-like phenotypes in rodents (Zheng et al., 2021). According to our previous study (Zhang et al., 2025), rats were exposed to CUMS daily from day 8 to day 28 for a total of 3 weeks. Each day, the rats were randomly exposed to two different stressors, which included water or food deprivation (24 h), swimming in cold water (12 °C) or hot (45 °C) water (5 min), continuous illumination (24 h), cage shaking (5 min) or tilting at a 45° angle (24 h), tail clamping (1 min), restraint (2 hours), noise (3 h), and wet bedding (24 h). The same stressor was not applied on two consecutive days to prevent the rats from predicting the stressor.

### 2.4 Group intervention

In addition to CUMS exposure, the CUMS + TPM group received daily TPM-like manipulations from day 1 to day 28. In the present study, the intervention was operationalized as standardized TPM-like manipulations applied along the dorsal midline, rather than as a direct reproduction of the clinical pediatric procedure. The intervention was applied 30 min before stressor exposure during the CUMS protocol, based on previous studies (Wu et al., 2024; Zhang et al., 2025). To ensure consistency, all manipulations were performed by the same trained operator. TPM-like manipulations were operationally defined as a standardized dorsal midline mechanical stimulation paradigm in rats, adapted from the main operational features of pediatric back stroking and spine-pinching manipulations. Following our prior protocol (Zhang et al., 2025). First, the rat was gently held, and the dorsal midline was stroked from the shoulder region to the sacral region five times using the fingers (pressure: ~5 N; speed: ~5 cm/s; Pressure Profile System, Inc., Hawthorne, CA, USA). Second, spine-pinching manipulation was applied by pinching and gently lifting the skin over the sacral midline, then twisting and pushing it cranially along the dorsal midline toward the cervicothoracic region for 15 repetitions (days 1–14) or 20 repetitions (days 15–28) (pressure: ~12 N; ~10 s/repetition). Third, the initial back stroking step was repeated. The selected force parameters were determined according to the clinical principle that, in pediatric TPM, spine-pinching intensity should be adjusted to a moderate level of stimulation tolerable to the child (Liang et al., 2020; Tong et al., 2022). In the present study, this principle was translated to the rat model by adjusting manipulation intensity according to the animals’ responses, with back stroking providing gentle stimulation and spine-pinching providing moderate stimulation. Throughout the procedure, no rats exhibited struggling or vocalization. The applied forces were quantified using a pressure measurement system to ensure standardization and reproducibility across sessions. Rats in the other groups were only gently held a few times as handling controls.

The use of a rodent model to investigate mechanisms underlying manual stimulation is supported by established cross-species similarities in cutaneous somatosensory innervation. Rodent dorsal skin contains low-threshold mechanoreceptors, including C-tactile-like afferents responsive to gentle stroking velocities within the range applied in the present protocol (Löken et al., 2009; Vrontou et al., 2013). Moreover, the rat paravertebral region shares analogous connective tissue organization and segmental sensory innervation with the human back (Corey et al., 2011). Previous studies from our team showed that TPM-like manipulations exerted an antidepressant effect in adolescent rats (Wu et al., 2024; Zhang et al., 2025).

In addition to CUMS exposure, the CUMS + FLX group received fluoxetine (H19980139, SIYAO, Changzhou, China; dissolved in 10% DMSO, 1 mg/mL, 10 mg/kg, i.p., once daily) 30 min before CUMS exposure. The other groups were given the same amount injections of DMSO instead.

### 2.5 Behavioral tests

The rats were weighed every other day. Behavioral tests were conducted after the 3-week modeling period, including the SPT, OFT, and MWM test.

SPT is considered a classically behavioral test for assessing anhedonia, a primary depression-like behavior in rodents (Jia et al., 2021; Gao et al., 2022). Briefly, each rat was provided with two 150 mL bottles of 1% sucrose water during the first 24 h for acclimatization. In the second 24 h, one bottle contained 1% sucrose water and the other pure water. In the third 24 h, no water was provided. In the fourth 24 h, the same setup as in the second 24 h was used, with the bottle positions exchanged every 12 h. The liquid consumption during the fourth 24 h was recorded. This procedure, including the 24-h water deprivation period, was applied identically to all groups. sucrose preference percentage (SPP) (%) = [Consumption of sucrose water/Total consumption of sucrose and pure water] × 100%.

OFT is a classically behavioral test used to evaluate both anxiety-like and depression - like behaviors in rodents (Kraeuter et al., 2019; Cao et al., 2023). Assessment typically involves measuring the time spent and distance traveled in the center zone. Their significance is based on the murine innate thigmotaxis, an aversion to open, exposed areas, which conflicts with the drive to explore a novel environment. Consequently, a reduced activity (both spent time and traveled distance) in center zone is interpreted as an increase in anxiety level (Zhang et al., 2023). The open field is a square arena (50 cm × 50 cm × 50 cm), with a digital camera (Topscan camera system, Cleve Sys, USA) positioned directly above it. Each rat was tested for 5 min after 2 h of acclimation before the test.

MWM test is a classical paradigm for assessing spatial learning and memory in rodents (Li et al., 2021; Wu et al., 2024). Cognitive impairment, including spatial memory deficits, is recognized as a core symptom of major depressive disorder (Rock et al., 2014; Darcet et al., 2016). Briefly, a dark circular pool (1.6 m in diameter) was filled with water at approximately 25 °C to a depth of 26 cm. A platform, 12 cm in diameter and 24 cm in height, was positioned in the center of a designated quadrant within the pool. The trajectory of each rat was recorded by Topscan camera system (Cleve Sys, USA). After an adaptation swim on the first test day, each rat underwent four trials per day for the following 4 days. In each trial, the rat began from a different quadrant and swam freely to locate the submerged platform within 60 s. The daily escape latency was averaged across the four trials as the time spent finding the platform. On the following day, the times of crossing the platform-located quadrant was recorded within 60 s after the platform was removed the platform.

### 2.6 Hematoxylin and eosin (HE) staining

Liver tissues were fixed with 4% paraformaldehyde, embedded in paraffin, and sectioned into 6-μm-thick slices. The sections were routinely dewaxed, rehydrated, and then stained with hematoxylin and counterstained with eosin. After dehydration and clearing, the sections were mounted with neutral balsam. Finally, images were observed and captured using a light microscope (DP70, Olympus, Tokyo, Japan). The histological inflammation scoring system was adapted from the literature (Chiu et al., 2024; Kim et al., 2025). For histopathological evaluation, five non-overlapping fields per animal were randomly selected and imaged at 200× magnification. All images were anonymized and randomized before analysis. Two independent investigators, blinded to group allocation, independently quantified inflammatory foci. An inflammatory focus was defined as a cluster of at least five inflammatory cells. The mean number of inflammatory foci per field was calculated for each animal, and the final value was obtained by averaging the results of the two investigators. The average inflammatory foci count was then converted into a 4-point inflammation score as follows: 0, < 0.5 foci per field; 1, 0.5–1.0 foci per field; 2, 1.0–2.0 foci per field; and 3, > 2.0 foci per field.

### 2.7 Quantitative reverse transcription polymerase chain reaction (qRT-PCR) analysis

For qRT-PCR analysis, four animals were randomly selected from each group. The total RNA was extracted from the hippocampus and liver using a Trizol reagent following the kit protocol (Invitrogen Life Technologies, Carlsbad, CA, USA). cDNA was synthesized by reverse transcription, and gene expression was then measured by qRT-PCR. The mRNA expressions were normalized to GAPDH and then to the control, and 2−ΔΔCT was used for quantitative comparison. The primers are shown in **Table 1**.

**Table 1:** Primer sequences for the target genes.

Gene	Forward primer (5’-3’)	Reverse primer (5’-3’)
GHR	GAATGGACCCCGGAATGGAA	GGCGGATCAGGTTGCACTAT
IGF-1	GGTGGACGCTCTTCAGTTC	TCCTCAGATCACAGCTCCG
IGF-1R	TTAACATCCGGCGAGGCAAT	GGAGACCAAGGCATGGGAAT
TNF-α	CCACCACGCTCTTCTGTC	GCTACGGGCTTGTCACTC
Il-1β	GAGAGCATCCAGCTTCAAA	TCATCATCCCACGAGTCA
GAPDH	CTCTCTGCTCCTCCCTGTTC	CGATACGGCCAAATCCGTTC

GHR, growth hormone receptor; IGF-1, insulin-like growth factor-1; IGF-1R, insulin-like growth factor-1 receptor; TNF-α, tumor necrosis factor alpha; IL-1β, interleukin-1β; GAPDH, glyceraldehyde-3-phosphate dehydrogenase.

### 2.8 Western blotting (WB) assay

Hippocampal and liver tissues were homogenized in lysis buffer. For WB assay, five animals were randomly selected from each group. Hippocampal and liver tissues were homogenized in lysis buffer. The blots were blocked for 20 minutes at room temperature and incubated overnight at 4 °C with the following primary antibodies: GAPDH (1:10000, A19056, ABclonal), GHR (1:1000, DF8425, Affinity), IGF-1 (1:1000, A24744, ABclonal), and IGF-1R (1:2000, A0243, ABclonal). Membranes were then incubated for 1 h at room temperature with the corresponding horseradish peroxidase-conjugated secondary antibodies: anti-rabbit IgG (1:2000, 7074S, Cell Signaling Technology) or anti-mouse IgG (1:2000, 7076S, Cell Signaling Technology). Band intensities were quantified using ImageJ software (NIH, Bethesda, MD, USA), normalized to GAPDH, and then expressed relative to the control group.

### 2.9 Enzyme-linked immunosorbent assay (ELISA)

After sample collection, plasma IGF-1 and hepatic L-aspartate concentrations were measured using ELISA kits (JX-1738A1 and JX-50365A1, Junxing Biotechnology, Jiangsu, China). Before ELISA, liver homogenates were quantified by Bicinchoninic Acid assay and diluted to the same total protein concentration, whereas plasma samples were measured directly according to the manufacturer’s instructions. Plasma ALT and AST concentrations were measured by using ELISA kits (CSB-E13024r, CSB-E13023r, Huamei Biotechnology, Wuhan).

### 2.10 Metabolite extraction

Samples for metabolomic analysis (n=5 per group) were randomly selected from the respective experimental groups to minimize selection bias. 50 mg liver tissue was added to a 2 mL centrifuge tube and a 6 mm diameter grinding bead was added. 400 μL of extraction solution (methanol: water = 4:1 (v:v) containing four internal standards (0.02 mg/mL L-2-chlorophenylalanine, etc.) were used for metabolite extraction. Samples were ground by the Wonbio-96c (Shanghai wanbo biotechnology co., LTD) frozen tissue grinder for 6 min (-10 °C, 50 Hz), followed by low-temperature ultrasonic extraction for 30 min (5 °C, 40 kHz). The samples were left at -20 °C for 30 min, centrifuged for 15 min (4 °C, 13000 g), and the supernatant was transferred to the injection vial for LC-MS/MS analysis. Meanwhile, 20 μL of every liver sample was used to prepare a pooled sample serving as the quality control (QC).

### 2.11 Ultra-performance liquid chromatography-tandem mass spectrometry instruments and conditions

The LC-MS/MS analysis of sample was conducted on a SCIEX UPLC-Triple TOF 6600 system equipped with an ACQUITY HSS T3 column (100 mm × 2.1 mm i.d., 1.8 μm; Waters, USA) at Majorbio Bio-Pharm Technology Co. Ltd. (Shanghai, China). The mobile phases consisted of 0.1% formic acid in water: acetonitrile (95:5, v/v) (solvent A) and 0.1% formic acid in acetonitrile: isopropanol: water (47.5:47.5, v/v) (solvent B). The column temperature was 45°C. The gradient used was: 0% (B), 0– 0.2min; 25% (B), 0.2–3 min; 100% (B), 3–9 min; 100% (B), 9–10 min; 0% (B), 10–10.1 min; 0% (B), 10.1–12 min. The flow rate was 0.40 mL/min. The injection volume was 10 μL.

The UPLC system was coupled to a SCIEX UPLC-Triple TOF 6600 Mass Spectrometer equipped with an electrospray ionization source operating in positive mode and negative mode. The optimal conditions were set as followed: source temperature at 500 °C; spray gas flow rate at 50 psi; Aux gas flow rate at 50 psi; curtain gas flow rate at 35 psi; ion-spray voltage floating at -4500V in negative mode and 5500V in positive mode, respectively; Normalized collision energy, 20-40-60V rolling for MS/MS. Data acquisition was performed with the Data Dependent Acquisition (DDA) mode. The detection was carried out over a mass range of 50–1200 m/z.

### 2.12 Data processing and metabolite identification for metabolomics analysis

Raw data were processed using Progenesis QI v3.0 (Waters Corporation, USA) for baseline filtering, peak picking, retention time correction, and peak alignment. Metabolite identification was performed by searching against public and in-house databases, including HMDB (http://www.hmdb.ca/) and Metlin (https://metlin.scripps.edu/). The resulting data matrix was preprocessed as follows: features with more than 20% missing values were removed in any group. Remaining missing values were imputed with the minimum value observed across all samples. To ensure data quality, only features with a relative standard deviation (RSD) ≤ 30% in the QC samples were retained for further analysis. The data were then normalized by total sum. Multivariate statistical analysis was performed using the “ropls” package in R (Version 1.6.2). Unsupervised Principal Component Analysis (PCA) was initially used to visualize the overall distribution and clustering of the samples. Subsequently, supervised Orthogonal Partial Least Squares Discriminant Analysis (OPLS-DA) was employed to maximize the separation between groups and identify differential metabolites. Metabolites with a Variable Importance in Projection (VIP) > 1.0 from the OPLS-DA model and a p-value < 0.05 (from a two-tailed Student’s t-test) were considered significantly different.

The differential metabolites between groups were annotated for metabolic pathways using the KEGG database (https://www.kegg.jp/kegg/pathway.html), allowing for the identification of pathways in which the differential metabolites are involved. Pathway enrichment analysis was conducted using the Python packages “scipy.stats” (https://docs.scipy.org/doc/scipy/).

### 2.13 Statistical analysis

Statistical analyses and graphing of data, except metabolomics data, were performed using SPSS 25.0 and GraphPad Prism 9.0, respectively. G*Power 3.1 was used for sample size estimation based on the primary endpoint and for *post hoc* calculations of Cohen’s f for the main outcomes. Based on effect sizes derived from our previous study (Zhang et al., 2025), a conservative Cohen’s f = 0.8 was assumed for the primary endpoint, SPP, and Cohen’s f = 1.0 for molecular endpoints. With α = 0.05, power = 0.80, and a one-way ANOVA with 4 groups, the required total sample sizes were estimated as 24 (n = 6) and 16 animals (n = 4), respectively. The Shapiro–Wilk test was used to assess normality of data distribution. For behavioral outcomes and other continuous variables that followed a normal distribution, one-way analysis of variance (ANOVA) was used for comparisons among groups. When homogeneity of variance was met, LSD was used for *post hoc* multiple comparisons; when normality was met but variances were unequal, Dunnett’s T3 test was applied. For non-normally distributed data, the Kruskal–Wallis H test was used for overall group comparisons, followed by pairwise Mann–Whitney U tests with Bonferroni correction when appropriate. Specifically, the number of platform crossings in the MWM probe trial did not meet normality assumptions and was therefore analyzed using the Kruskal–Wallis H test followed by Bonferroni-adjusted *post hoc* pairwise comparisons. Escape latency in the MWM acquisition phase was analyzed using two-way repeated-measures ANOVA. For correlations, Pearson’s correlation coefficient was used when both variables were normally distributed; otherwise, Spearman’s rank correlation was used. Inter-rater reliability for histological inflammatory assessment was evaluated using the intraclass correlation coefficient (ICC) based on a two-way random-effects model with absolute agreement, and both single-measure and average-measure ICCs with 95% confidence intervals were reported. Normally distributed data were expressed as mean ± standard error of the mean (SEM). *P* value <0.05 was considered statistically significant.

## 3 Results

### 3.1 TPM-like manipulations prevented CUMS-induced depression-related behaviors in adolescent female rats

**Figure 2:**
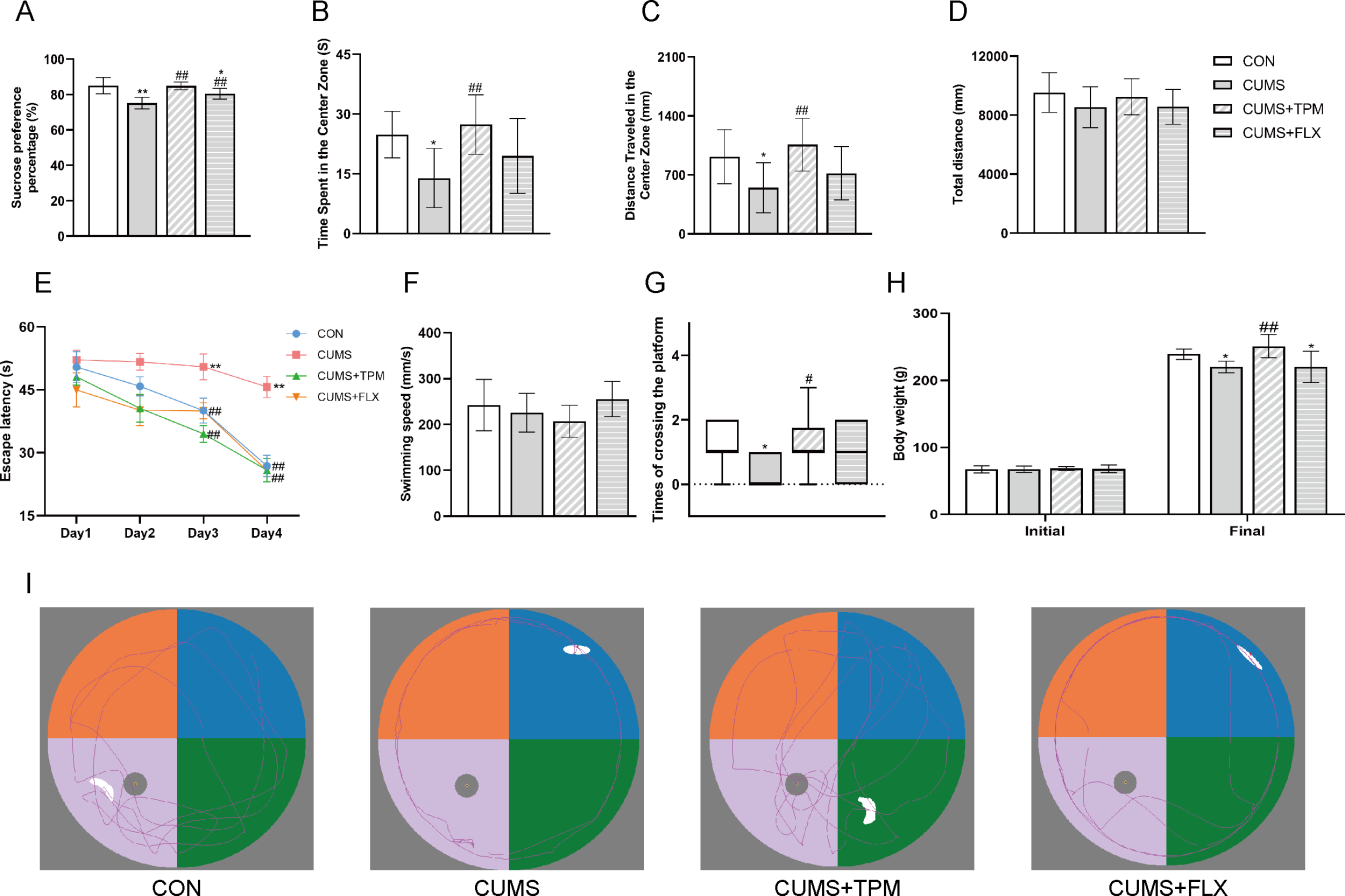
TPM-like manipulations prevented CUMS-induced depression-related behaviors in adolescent female rats. **(A)** Sucrose preference percentage in the SPT. **(B)** Time spent in the center zone of the OFT. **(C)** Traveled distance in the center zone of the OFT. **(D)** Total distance traveled in the OFT. **(E)** Escape latency during the acquisition trials of the MWM. **(F)** Swimming speed on day 4 of the acquisition phase in the MWM. **(G)** Times of crossing the platform in the MWM. **(H)** The initial and final body weights of early adolescent rats. **(I)** Typical trajectories of spatial exploration in the MWM. Platform crossing data in the MWM probe trial were analyzed using the Kruskal–Wallis H test followed by pairwise Mann–Whitney *U* tests with Bonferroni correction. Other data were analyzed using one-way ANOVA followed by LSD *post hoc* tests. Platform crossing data are presented as interquartile ranges, whereas the other data are presented as mean ± SEM (n = 7–8). *P < 0.05, **P < 0.01 vs. the CON group. ^#^*P* < 0.05, ^##^*P* < 0.01 vs. the CUMS group. CON, control; CUMS, chronic unpredictable mild stress; TPM, traditional pediatric massage-like manipulations; FLX, fluoxetine; SPT, sucrose preference test; OFT, open field test; MWM, Morris water maze.

SPT, OFT, and MWM tests to assess depression-related behavioral changes in adolescent female rats exposed to CUMS. As the primary outcome measure selected for *a priori* power analysis, SPP in the SPT showed a significant group effect by one-way ANOVA (F _3, 26_ = 13.606, *P* < 0.01, η² = 0.611, Cohen’s f = 1.216; **Figure 2A**). Similarly, in the OFT, significant group differences were found in both time spent in the center zone (F _3, 26_ = 4.626, *P* < 0.01, η² = 0.348; **Figure 2B**) and distance traveled in the center zone (F _3, 26_ = 3.813, *P* < 0.05, η² = 0.306; **Figure 2C**), whereas no significant group difference was found in total travel distance (F _3, 26_ = 1.129, *P* > 0.05, η² = 0.115; **Figure 2D**). Compared with the CON group, the CUMS group showed significantly lower SPP in the SPT (*P* < 0.01; **Figure 2A**) and less time spent and distance traveled in the center zone in the OFT (*P* < 0.05, *P* < 0.05; **Figure 2C**). These changes were attenuated in the CUMS + TPM group (*P* < 0.01, *P* < 0.01, *P* < 0.01; **Figures 2A–C**), whereas partial improvement was observed in the FLX group (*P* < 0.01, *P* > 0.05, *P* > 0.05; **Figures 2A–C**). Two-way repeated-measures ANOVA of escape latency in the MWM revealed a significant main effect of time (F _3, 78_ = 31.394, *P* < 0.01, η² = 0.547), indicating an overall learning effect across training days. A significant group effect was also observed for escape latency across the acquisition phase (F _3, 26_ = 15.874, *P* < 0.01, η² = 0.647). Further one-way ANOVA showed that CUMS exposure significantly prolonged escape latency on the 3rd (F _3, 26_ = 6.687, *P* < 0.01, η² = 0.436; **Figure 2E**) and 4th training days (F _3, 26_ = 15.421, *P* < 0.01, η² = 0.640; **Figure 2E**), suggesting impaired spatial learning performance in CUMS-exposed early adolescent rats. Both TPM-like manipulations and FLX were associated with shorter escape latency on training days 3 and 4 compared with the CUMS group (all *P* < 0.01; **Figure 2E**). No significant difference in swimming speed on day 4 was observed among groups (F _3, 26_ = 1.737, *P* > 0.05, η² = 0.167; **Figure 2F**), suggesting that the differences in escape latency were unlikely to be explained simply by impaired swimming ability. In the probe trial on day 5, platform crossings were analyzed using the Kruskal–Wallis H test followed by Bonferroni-adjusted *post hoc* pairwise comparisons because normality assumptions were not met. A significant overall group difference was found [H(3) = 10.486, *P* < 0.05; **Figure 2G**]. The CUMS group showed fewer platform crossings than the CON group (adjusted *P* < 0.05; **Figure 2G**). TPM-like manipulations increased the number of platform crossings compared with the CUMS group (adjusted *P* < 0.05; **Figure 2G**), whereas FLX did not (adjusted *P* > 0.05; **Figure 2G**).

Early adolescent rats undergo rapid development, which can be impaired by CUMS. Body weight is a key index to evaluate the development status in rodents (Borsini et al., 2023). One-way ANOVA revealed significant differences of body weight after different treatments among groups (F _3, 26_ = 8.523, *P* < 0.01, η² = 0.459; **Figure 2H**). CUMS exposure caused a significant decrease in body weight (*P* < 0.05; **Figure 2H**), which was significantly prevented by TPM-like manipulations (*P* < 0.01; **Figure 2H**) but not by FLX (*P* > 0.05; **Figure 2H**).

### 3.2 TPM-like manipulations reduced inflammatory marker expression and increased IGF-1 and IGF-1R expression in the hippocampus

**Figure 3:**
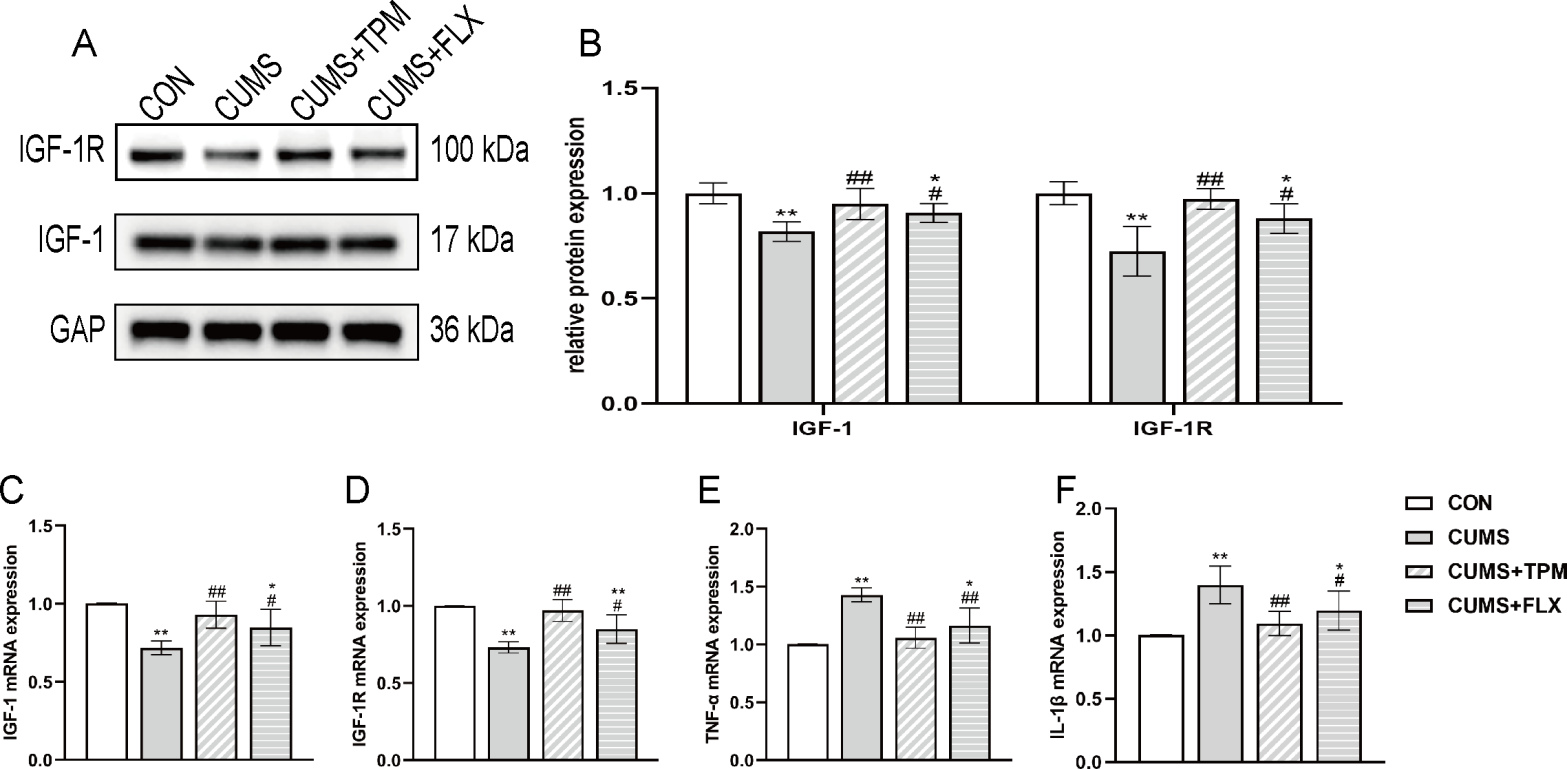
TPM-like manipulations increased the expression of IGF-1 and IGF-1R as well as the inflammation in the hippocampus of CUMS-exposed adolescent female rats. **(A)** Representative bands of IGF-1 and IGF-1R detected by WB. **(B)** Protein expressions of IGF-1 and IGF-1R in the hippocampus detected by WB. **(C)** IGF-1 mRNA expression in the hippocampus assessed by qRT-PCR. **(D)** IGF-1R mRNA expression in the hippocampus assessed by qRT-PCR. **(E)** TNF-α mRNA expression in the hippocampus assessed by qRT-PCR. **(F)** IL-1β mRNA expression in the hippocampus assessed by qRT-PCR. All data were analyzed using one-way ANOVA followed by LSD *post hoc* tests. Data are presented as mean ± SEM (n = 4 for qRT-PCR; n = 5 for WB). ^*^P < 0.05, ^**^P < 0.01 vs. the CON group. ^#^*P* < 0.05, ^##^*P* < 0.01 vs. the CUMS group. CON, control; CUMS, chronic unpredictable mild stress; TPM, traditional pediatric massage-like manipulations; FLX, fluoxetine; IGF-1, insulin-like growth factor-1; IGF-1R, insulin-like growth factor-1 receptor; TNF-α, tumor necrosis factor alpha; IL-1β, interleukin-1β; WB, Western blotting; qRT-PCR, Quantitative reverse transcription Polymerase Chain Reaction.

Neuroinflammation has been implicated in depression (Zhang et al., 2025). As a neuroprotective factor, IGF-1 has been reported to exert anti-inflammatory effects through binding to its receptor (IGF-1R) (Huo et al., 2014; Sun et al., 2020). We examined inflammatory marker expression as well as IGF-1 and IGF-1R expression in the hippocampus, a brain region critically involved in mood regulation and cognition. One-way ANOVA revealed significant group effects on IGF-1 Mrna (F _3, 12_ = 10.267, *P* < 0.01, η² = 0.720; **Figure 3C**), IGF-1R mRNA(F _3, 12_ = 16.351, *P* < 0.01, η² = 0.803; **Figure 3D**), IGF-1 protein (F _3, 16_ = 9.721, *P* < 0.01, η² = 0.646; **Figure 3B**), and IGF-1R protein (F _3, 16_ = 12.951, *P* < 0.01, η² = 0.708; **Figure 3B**).Compared with the CON group, the CUMS group showed lower IGF-1 mRNA and protein expression (*P* < 0.01, *P* < 0.01; **Figures 3B, C**) and lower IGF-1R mRNA and protein expression (*P* < 0.01, *P* < 0.01; **Figures 3B, D**) in the hippocampus. Compared with the CUMS group, TPM-like manipulations were associated with higher IGF-1 mRNA and protein levels (*P* < 0.01, *P* < 0.01; **Figures 3B, C**) and higher IGF-1R mRNA and protein levels (*P* < 0.01, *P* < 0.01; **Figures 3B, D**). One-way ANOVA revealed significant group effects on TNF-α mRNA (F _3, 12_ = 16.292, *P* < 0.01, η² = 0.803; **Figure 3E**) and IL-1β mRNA (F _3, 12_ = 8.269, *P* < 0.01, η² = 0.674; **Figure 3F**), both of which are typical inflammatory markers. Compared with the CON group, the CUMS group showed higher TNF-α and IL-1β mRNA expression (*P* < 0.01, *P* < 0.01; **Figures 3E, F**). TPM-like manipulations significantly suppressed the upregulation of mRNA expressions of TNF-α and IL-1β (*P* < 0.01, *P* < 0.01; **Figure 3** E, F). FLX also had a similar effect, that is, reversed the decreases in the gene and protein expressions of IGF-1 (*P* < 0.05, *P* < 0.05; **Figures 3B, C**) and IGF-1R (*P* < 0.01, *P* < 0.05; **Figures 3B, D**) and decreased the gene expressions of TNF-α and IL-1β (*P* < 0.01, *P* < 0.05; **Figures 3E, F**).

### 3.3 TPM-like manipulations increased hepatic GHR and IGF-1 expression in CUMS-exposed adolescent female rats

Circulating IGF-1 is considered an important source of IGF-1 for the central nervous system, including the hippocampus (Ivan et al., 2023). IGF-1 produced in the liver is the principal source of circulating IGF-1 (Pharaoh et al., 2020), which is under GHR-mediated GH regulation. We further measured hepatic GHR and IGF-1 expression and plasma IGF-1 concentration to explore liver-brain axis-related changes associated with TPM-like manipulations.

**Figure 4:**
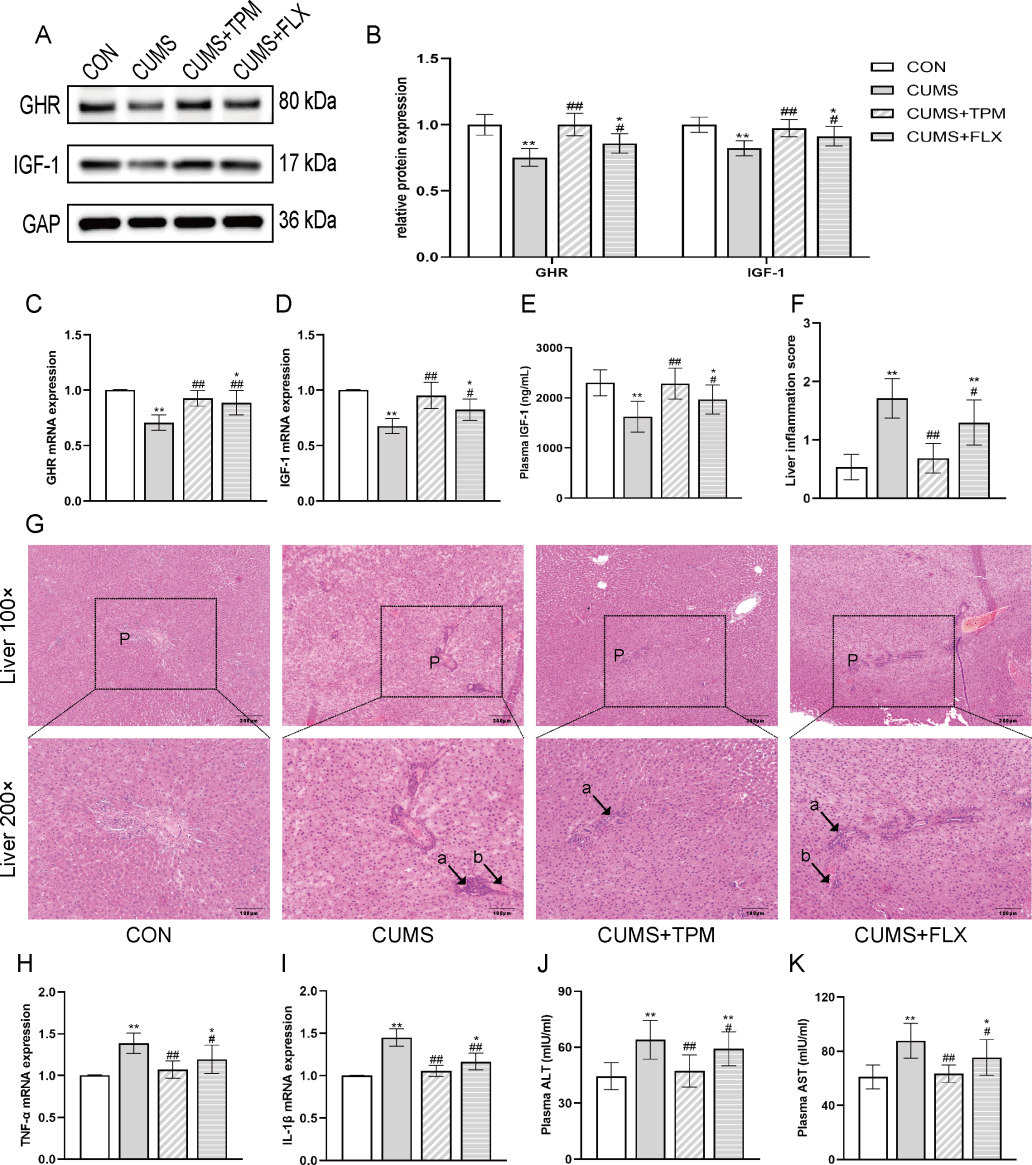
TPM-like manipulations maintained GHR/IGF-1 signaling activity in the liver in CUMS-exposed adolescent female rats. **(A)** Representative bands of GHR and IGF-1 detected by WB. **(B)** Protein expressions of GHR and IGF-1 in the liver detected by WB. **(C)** GHR mRNA expression in the liver assessed by qRT-PCR. **(D)** IGF-1 mRNA expression in the liver assessed by qRT-PCR. **(E)** Plasma IGF-1 levels in the liver assessed by ELISA. **(F)** Liver inflammation score. **(G)** Representative HE-stained liver sections. Representative low-magnification images (100×, scale bar = 200 μm) and corresponding high-magnification insets (200×, scale bar = 100 μm) are shown. “P” indicates the portal area, Arrow “a” indicates inflammatory cell infiltration, and arrow “b” indicates hemorrhage. **(H)** TNF-α mRNA expression in the liver assessed by qRT-PCR. **(I)** IL-1β mRNA expression in the liver assessed by qRT-PCR. **(J)** Plasma ALT levels assessed by ELISA. **(K)** Plasma AST levels assessed by ELISA. All data were analyzed using one-way ANOVA followed by LSD *post hoc* tests. Data are presented as mean ± SEM (n = 4 for qRT-PCR; n = 5 for western blotting; n = 7–8 for ELISA and liver inflammation scoring). ^*^P < 0.05, ^**^P < 0.01 vs. the CON group. ^#^*P* < 0.05, ^##^*P* < 0.01 vs. the CUMS group. CON, control; CUMS, chronic unpredictable mild stress; TPM, traditional pediatric massage-like manipulations; FLX, fluoxetine; GHR, growth hormone receptor; IGF-1, insulin-like growth factor-1; TNF-α, tumour necrosis factor alpha; IL-1β, interleukin-1β; ALT, alanine aminotransferase; AST, aspartate aminotransferase; qRT-PCR, Quantitative reverse transcription Polymerase Chain Reaction; ELISA, Enzyme-linked immunosorbent assay.

One-way ANOVA revealed significant group effects on hepatic GHR mRNA (F _3, 12_ = 11.452, *P* < 0.01, η² = 0.741; **Figure 4C**), hepatic IGF-1 mRNA (F _3, 12_ = 12.298, *P* < 0.01, η² = 0.755; **Figure 4D**), hepatic GHR protein (F _3, 16_ = 12.694, *P* < 0.01, η² = 0.704; **Figure 4B**), and hepatic IGF-1 protein (F _3, 16_ = 7.54, *P* < 0.01, η² = 0.586; **Figure 4B**). Compared with the CON group, the CUMS group showed lower hepatic GHR mRNA and protein expression (*P* < 0.01, *P* < 0.01; **Figures 4B, C**) and lower hepatic IGF-1 mRNA and protein expression (*P* < 0.01, *P* < 0.01; **Figures 4B, D**) in the liver. Compared with the CUMS group, TPM-like manipulations were associated with higher hepatic GHR mRNA and protein expression (*P* < 0.01, *P* < 0.01; **Figures 4B, C**) and higher hepatic IGF-1 mRNA and protein expression (*P* < 0.01, *P* < 0.01; **Figures 4B, D**). Consistent with the liver findings, one-way ANOVA revealed a significant group difference in plasma IGF-1 concentration (F _3, 26_ = 8.852, *P* < 0.01, η² = 0.505; **Figure 4E**) among groups. Compared with the CON group, the CUMS group showed a lower plasma IGF-1 concentration (*P* < 0.01; **Figure 4E**), whereas TPM-like manipulations were associated with a higher plasma IGF-1 concentration compared with the CUMS group (*P* < 0.01; **Figure 4E**).

FLX was also associated with higher hepatic GHR mRNA and protein expression (*P* < 0.05, *P* < 0.05; **Figures 4B, C**), higher hepatic IGF-1 mRNA and protein expression (*P* < 0.01, *P* < 0.05; **Figures 4B, D**), and higher plasma IGF-1 concentration (*P* < 0.05; **Figure 4E**).

### 3.4 TPM-like manipulations inhibited the liver inflammation in CUMS-exposed adolescent female rats

Because CUMS may induce liver inflammation (Mehranfard et al., 2020; Xu et al., 2022), we further examined hepatic inflammatory and injury-related changes. HE staining of liver tissues (**Figure 4G**) showed that the CON group had normal lobular architecture, with well-aligned hepatic cords, clearly defined sinusoids, and hepatocytes with uniform cytoplasm and intact nuclei. In contrast, the CUMS group showed obvious pathological changes, including disorganized hepatic cords, inflammatory cell infiltration, vascular congestion, and focal hemorrhage. The TPM-like manipulations group showed less obvious pathological change, whereas the FLX group still showed mild pathological change. One-way ANOVA revealed a significant group difference in liver histological inflammation scores (F _3, 26_ = 24.317, *P* < 0.01, η² = 0.737; **Figure 4F**). Compared with the CON group, the CUMS group showed a higher liver inflammation score (*P* < 0.01; **Figure 4F**). Compared with the CUMS group, TPM-like manipulations were associated with a lower liver inflammation score (*P* < 0.01; **Figure 4F**). FLX was also associated with a lower liver inflammation score (*P* < 0.05; **Figure 4F**). Inter-rater reliability for liver histological inflammation scoring was high, with a single-measure ICC of 0.881 (95% CI, 0.768–0.941) and an average-measure ICC of 0.937 (95% CI, 0.868–0.970).

Consistent with these findings, one-way ANOVA revealed significant group effects on hepatic TNF-α mRNA (F _3, 12_ = 8.220, *P* < 0.01, η² = 0.673; **Figure 4H**) and hepatic IL-1β mRNA (F _3, 12_ = 25.819, *P* < 0.01, η² = 0.866; **Figure 4I**). Compared with the CON group, the CUMS group showed higher hepatic TNF-α and IL-1β mRNA expression (*P* < 0.01, *P* < 0.01; **Figures 4H, I**). Compared with the CUMS group, TPM-like manipulations were associated with lower hepatic TNF-α and IL-1β mRNA expression (*P* < 0.01, *P* < 0.01; **Figures 4H, I**). One-way ANOVA revealed significant group differences in plasma ALT (F _3, 26_ = 17.503, *P* < 0.01, η² = 0.669; **Figure 4J**) and AST (F _3, 26_ = 10.106, *P* < 0.01, η² = 0.538; **Figure 4K**). Compared with the CON group, the CUMS group showed higher plasma ALT and AST concentrations (*P* < 0.01, *P* < 0.01; **Figures 4J, K**). Compared with the CUMS group, TPM-like manipulations were associated with lower plasma ALT and AST concentrations (*P* < 0.01, *P* < 0.01; **Figures 4J, K**). FLX was also associated with lower hepatic TNF-α mRNA (*P* < 0.05; **Figure 4H**), lower hepatic IL-1β mRNA (*P* < 0.01; **Figure 4I**), and lower plasma ALT and AST concentrations (*P* < 0.01, *P* < 0.01; **Figures 4J, K**).

### 3.5 TPM-like manipulations were associated with changes in hepatic amino acid metabolism in CUMS-exposed adolescent female rats, including higher L-Aspartic Acid levels

Several studies have suggested that CUMS may be associated with hepatic metabolic disturbances (Geng et al., 2020; Jia et al., 2023). We next examined whether hepatic metabolic alterations were associated with inflammatory markers, IGF-1-related indices, and depression-like behaviors. Therefore, non-targeted metabolomic profiling of liver tissue was performed by UPLC-MS/MS.

#### 3.5.1 Hepatic metabolomic profiling

**Figure 5:**
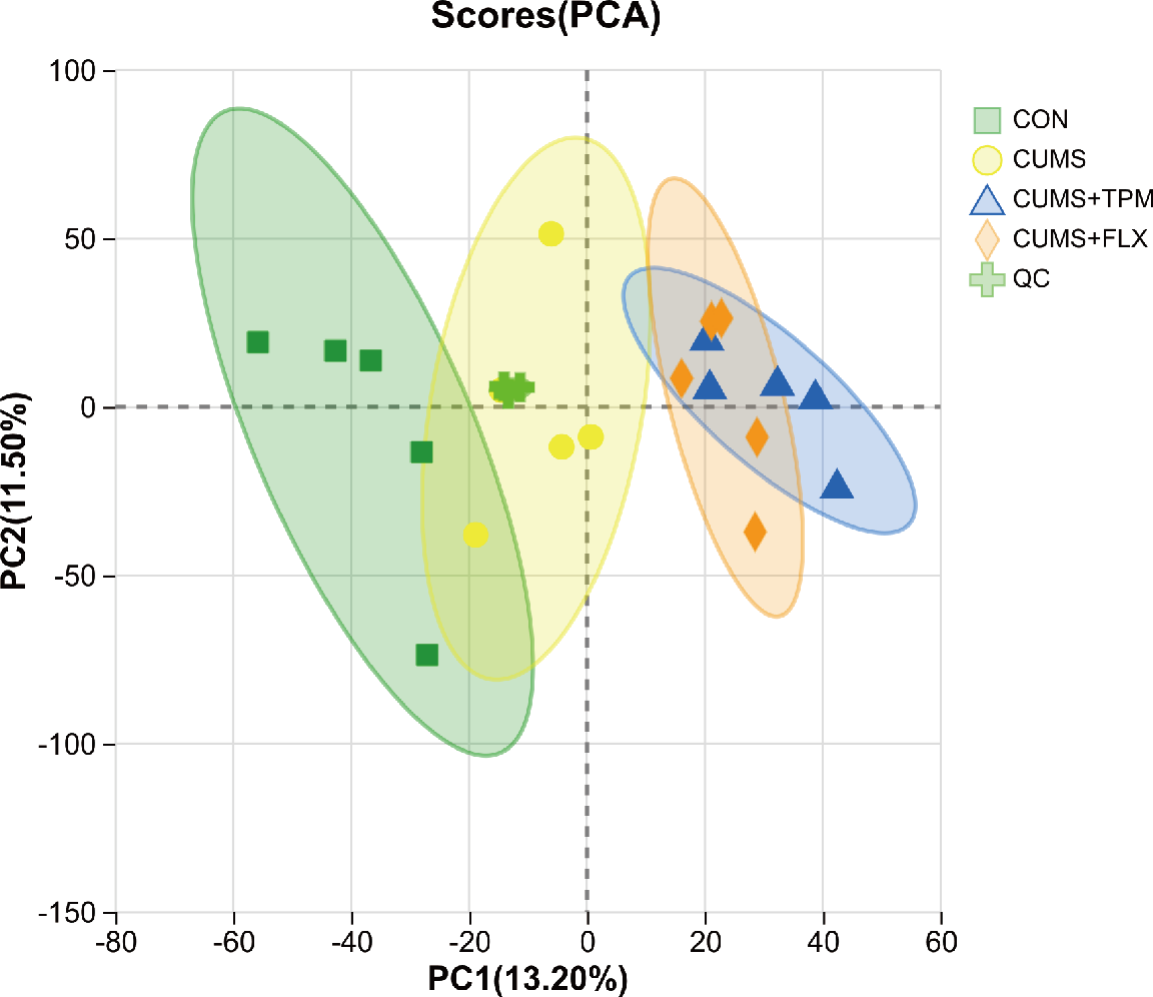
Hepatic metabolomic profiling. PCA score plot of liver samples from the CON, CUMS, CUMS + TPM, and CUMS + FLX groups (n = 5). CON, control; CUMS, chronic unpredictable mild stress; TPM, traditional pediatric massage-like manipulations; FLX, fluoxetine; PCA, principal component analysis.

PCA was first used to provide an overview of global metabolic variation among groups, with QC procedures applied to assess data quality. As shown in **Figure 5**, QC samples clustered relatively closely in the PCA score plot, suggesting acceptable analytical stability. PC1 and PC2 accounted for 13.2% and 11.5% of the total variance, respectively. The PCA plot showed a general trend of group-related metabolic variation. PLS-DA and OPLS-DA were additionally conducted as exploratory supervised analyses, and the corresponding score plots and permutation tests are presented in the **Supplementary Materials**. The PLS-DA permutation test yielded an R2Y of 0.622 and a Q2 of 0.35, indicating limited predictive performance. Accordingly, these multivariate models were interpreted cautiously and were used only as auxiliary tools for exploratory analysis.

Metabolites were putatively annotated by matching MS/MS data against public databases such as HMDB and Metlin. Differential metabolites were screened using the combined criteria of VIP > 1.0 and *P* < 0.05 in pairwise comparisons. By taking the union of the resulting differential metabolite lists, 22 candidate metabolites were retained for subsequent analyses. Among these candidate metabolites, L-Aspartic Acid was selected for further analysis because of its potential relevance to amino acid metabolism and liver-brain axis-related changes.

#### 3.5.2 Differential metabolites and pathway analysis in the liver

Compared with the CON group, the CUMS group showed lower relative abundances of putatively annotated metabolites including L-Aspartic Acid, L-Methionine, 15-Hete, 2-methoxy ketone-3-glucoside, Lpe (20:2), Lpc (20:2), and N-arachidonoyl ethanolamine, together with higher relative abundances of Val-Lys, valine, N-myristoyl methionine, GPETN (20:2/18:2), sebacic acid, and Stearoyl Ethanolamide. Compared with the CUMS group, the CUMS + TPM group showed higher relative abundances of putatively annotated metabolites including L-Aspartic Acid, 15-Hete, L-Proline, Gly-Glu, Farnesylcysteine, N-Arachidonoyl Threonine, Glycerol 2-Phosphate, and L-Serine, together with lower relative abundances of Val-Lys, Sebacic Acid, Pe (17:1/0:0), Tetradecanedioic Acid, Galactosylsphingosine, and Pseudouridine. Compared to CUMS group, the CUMS +FLX group showed lower relative abundances of N-Myristoyl Methionine, Sebacic Acid, Tetradecanedioic Acid, Galactosylsphingosine, and Pseudouridine, together with higher relative abundances of Gly-Glu, Farnesylcysteine, and Glycerol 2-Phosphate.The differential metabolites in detail are shown in **Table 2**.

**Table 2:** Differential metabolites in the liver.

Metabolite	Retention time (min)	Adducts	Formula	CUMS_VS_CON	CUMS + TPM_VS_CUMS	CUMS + FLX_VS_CUMS
N-Arachidonylethanolamine	8.548833333	M+FA-H	C22H39NO	↓**	↑	↓
L-Aspartic Acid	0.549983333	M-H	C4H7NO4	↓*	↑###	↑
15-Hete	7.5099	M-H	C20H32O3	↓*	↑##	↑
L-Methionine	1.163916667	M+H	C5H11NO2S	↓*	↑	↑
2-Methoxyestrone 3-Glucuronide	5.783166667	M+Na-2H	C25H32O9	↓*	↑	↓#
Lpe(20:2)	8.271016666669999	M-H	C25H48NO7P	↓*	↑	↓
Lpc(20:2)	8.221366666669999	M+FA-H	C28H54NO7P	↓*	↑	↑
L-Proline	0.60365	M+H	C5H9NO2	↓	↑##	↑
Gly-Glu	0.5988	M-H	C7H12N2O5	↓	↑##	↑#
Farnesylcysteine	6.701116666669999	M-H	C18H31NO2S	↓	↑##	↑#
N-Arachidonoyl Threonine	8.141366666669999	M-H	C24H39NO4	↓	↑##	↑
Glycerol 2-Phosphate	0.6768	M-H	C3H9O6P	↓	↑##	↑##
L-Serine	0.54015	M-H	C3H7NO3	↓	↑##	↑
Val-Lys	0.54755	M+K	C11H23N3O3	↑**	↓##	↓
N-Myristoyl Methionine	8.221366666669999	M-H	C19H37NO3S	↑**	↓	↓###
Gpetn(20:2/18:2)	10.53001667	M+Na	C43H78NO8P	↑**	↓	↑
Sebacic Acid	4.948883333	M-H	C10H18O4	↑*	↓##	↓#
Stearoyl Ethanolamide	8.93285	M+H	C20H41NO2	↑*	↓	↑
Pe(17:1/0:0)	7.887216667	M-H	C22H44NO7P	↑	↓#	↓
Tetradecanedioic Acid	6.581116666669999	M-H	C14H26O4	↑	↓###	↓##
Galactosylsphingosine	8.450516667	2M+FA-H	C24H47NO7	↑	↓#	↓##
Pseudouridine	1.17305	M+H	C9H12N2O6	↑	↓###	↓#

(n = 5) ^*^P < 0.05, ^**^P < 0.01 vs. the CON group. ^#^*P* < 0.05, ^##^*P* < 0.01, ^###^*P* < 0.001 vs. the CUMS group. CON, control; CUMS, chronic unpredictable mild stress; TPM, traditional pediatric massage-like manipulations; FLX, fluoxetine.

**Figure 6:**
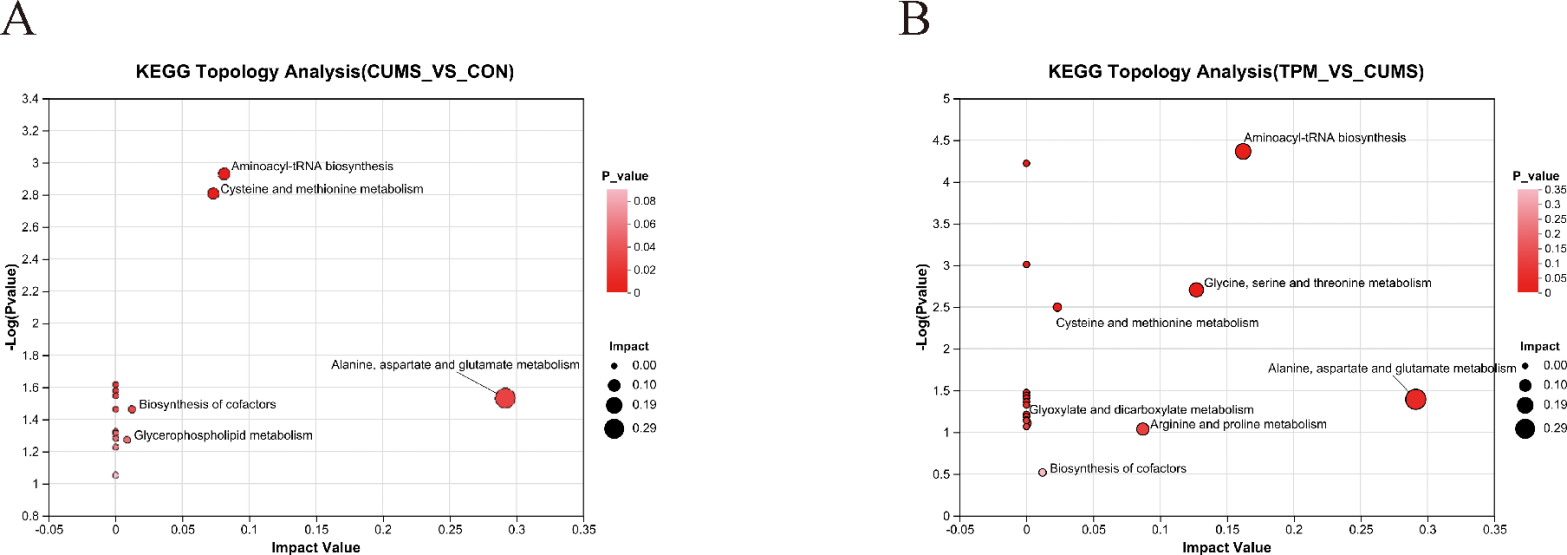
KEGG pathway enrichment analysis of differential metabolites in liver tissue. **(A)** Depression-associated metabolic pathways; **(B)** Traditional pediatric massage -like manipulation affected metabolic pathways associated with depression.

KEGG pathway analysis was then performed on the differential metabolites identified in the pairwise comparisons. Among the metabolites differing between the CON and CUMS groups, pathway enrichment analysis suggested enrichment of aspartate, alanine and glutamate metabolism, aminoacyl-tRNA biosynthesis, cysteine and methionine metabolism, and biosynthesis of cofactors (**Figure 6A**). These results suggest that CUMS exposure may be associated with alterations in amino acid-related metabolic pathways in the liver. Among the metabolites differing between the CUMS and CUMS + TPM groups, pathway enrichment analysis suggested enrichment of aspartate, alanine and glutamate metabolism, aminoacyl-tRNA biosynthesis, glycine, serine and threonine metabolism, and cysteine and methionine metabolism (**Figure 6B**), suggesting that TPM-like manipulations were associated with partial normalization of several amino acid-related metabolic alterations. In contrast, although several differential metabolites were observed in the CUMS + FLX group relative to the CUMS group, no pathway reached the preset significance threshold in the KEGG enrichment analysis.

#### 3.5.3 Hepatic metabolites were correlated with behavioral outcomes and IGF-1-related indices in the liver and hippocampus

To further examine whether hepatic metabolic changes were associated with depression-related behaviors and IGF-1-related indices in the liver and brain, correlation analyses were performed.

**Figure 7:**
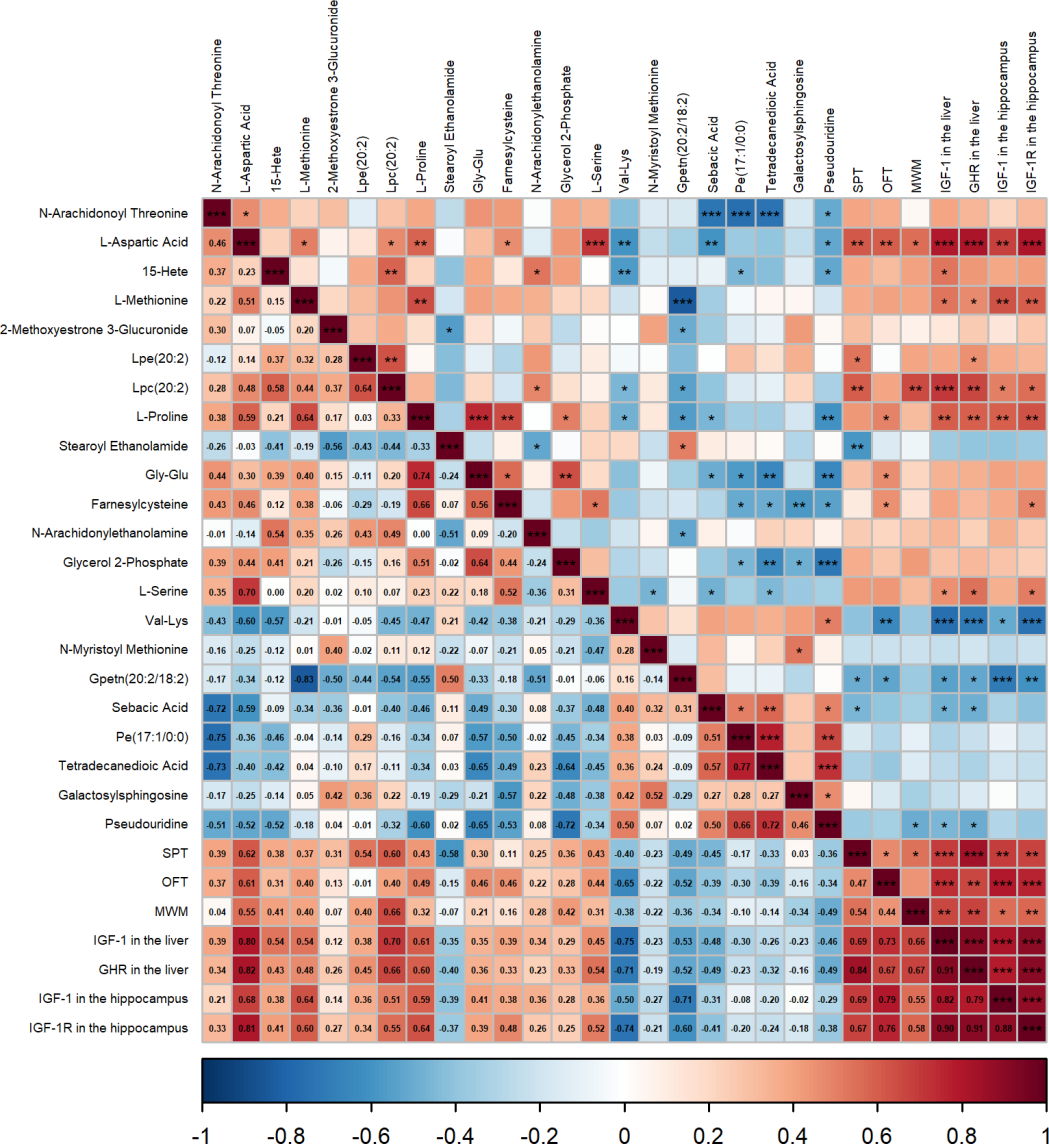
Spearman correlation matrix of hepatic metabolic changes, behavioral outcomes, and IGF-1-related protein expression in the liver and hippocampus. The heatmap illustrates the Spearman’s rank correlation coefficients (r) between the relative abundance of the 22 key differential hepatic metabolites and seven key outcome variables: behavioral parameters including sucrose preference percentage, the time spent in the center zone in the open field test, and the number of platform crossings in the Morris water maze probe trial, hepatic protein expression of growth hormone receptor and insulin-like growth factor 1, and the protein expression of insulin-like growth factor 1 and insulin-like growth factor 1 receptor in the hippocampus. The color scale indicates the strength and direction of the correlation, with red representing a positive correlation and blue representing a negative correlation. Asterisks denote the level of statistical significance. (n = 5). ^*^P < 0.05, ^**^P < 0.01, ^***^P < 0.001.

Spearman’s rank correlation analysis revealed a series of significant correlations among hepatic metabolites, behavioral outcomes, and IGF-1-related indices (**Figure 7**). In particular, several putatively annotated metabolites that were higher in the CUMS + TPM group, including L-Aspartic Acid, L-Proline, and Lpc(20:2), showed positive correlations with hepatic IGF-1 and GHR protein expression, as well as hippocampal IGF-1 and IGF-1R protein expression. Furthermore, L-Aspartic Acid was positively correlated with L-Methionine, Lpc(20:2), L-Proline, Farnesylcysteine, and L-Serine. These metabolites were also positively correlated with behavioral indices, including SPP in the SPT, time spent in the center zone in the OFT, and the number of platform crossings in the MWM.

Moreover, several putatively annotated metabolites that were higher in the CUMS group and lower in the TPM-like manipulations group, such as Val-Lys, Gpetn (20:2/18:2), Sebacic Acid, and Pseudouridine, showed negative correlations with behavioral indices, hepatic IGF-1 and GHR protein expression, and hippocampal IGF-1 and IGF-1R protein expression.

In sum, these correlation analyses suggested that hepatic metabolites and IGF-1-related indices were associated with behavioral changes and liver-brain axis-related alterations. Moreover, TPM-like manipulations were associated with changes in amino acid metabolism and GHR/IGF-1-related indices.

### 3.6 TPM-like manipulations increased the concentration of L-aspartic acid

**Figure 8:**
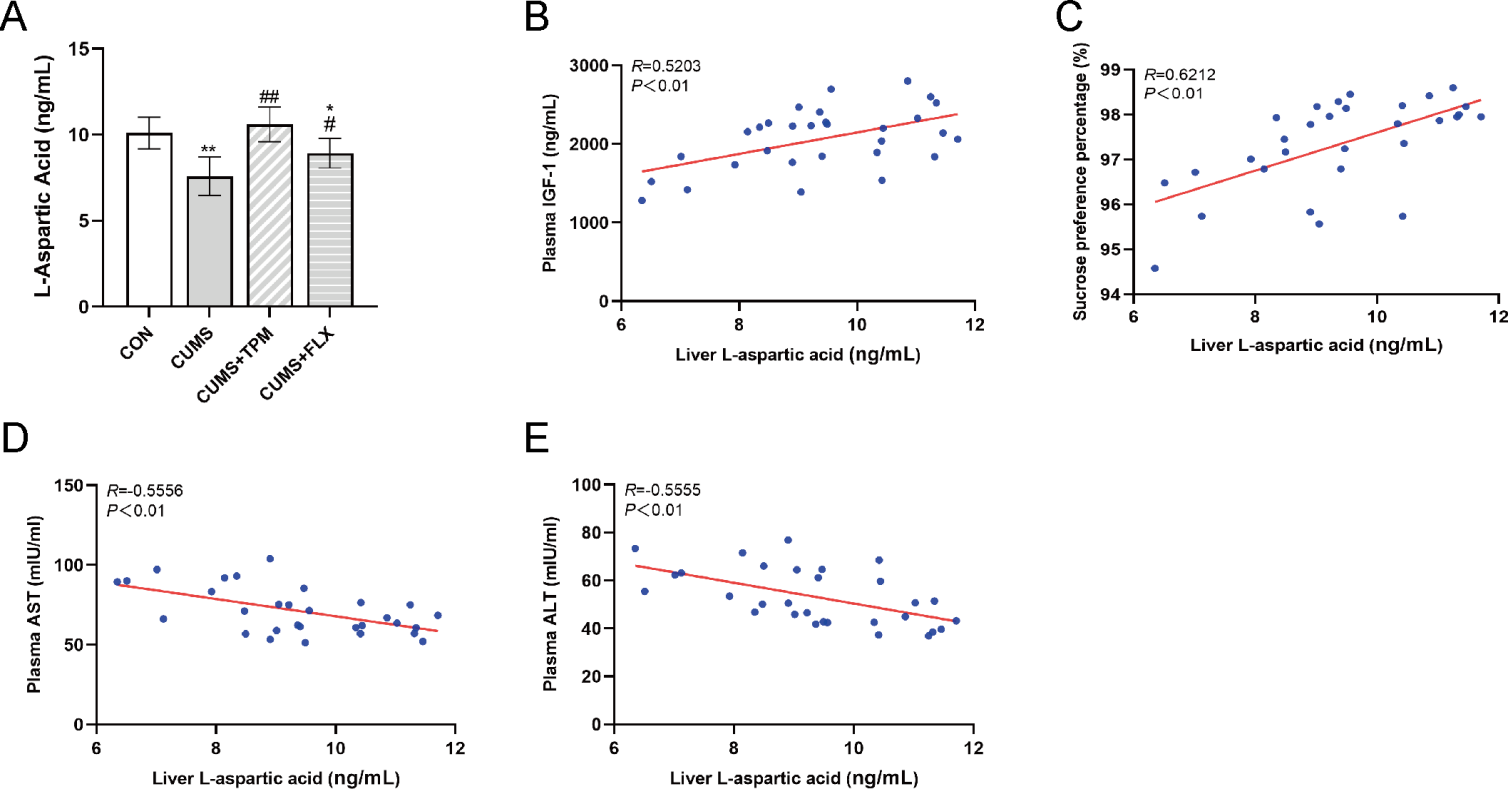
Correlation analysis of hepatic L-aspartic acid changes with circulating IGF-1 concentration, liver injury-related indices, and depression-related behaviors. **(A)** L-Aspartic Acid concentration in the liver assessed by ELISA **(B)** Correlation analysis between liver L-Aspartic Acid and plasma IGF-1. **(C)** Correlation analysis between liver L-Aspartic Acid and sucrose preference percentage in the sucrose preference test. **(D)** Correlation analysis between liver L-Aspartic Acid and plasma AST. **(E)** Correlation analysis between liver L-Aspartic Acid and plasma ALT. Hepatic L-Aspartic Acid data were analyzed using one-way ANOVA followed by LSD *post hoc* tests. Pearson correlation analysis was performed for panels B–E. Data are presented as mean ± SEM (*n* = 7–8 for ELISA; *n* = 28 for Pearson correlation analysis). ^*^P < 0.05, ^**^P < 0.01 vs. the CON group. ^#^*P* < 0.05, ^##^*P* < 0.01 vs. the CUMS group. CON, control; CUMS, chronic unpredictable mild stress; TPM, traditional pediatric massage-like manipulations; FLX, fluoxetine; IGF-1, insulin-like growth factor-1; AST, aspartate aminotransferase; ALT, alanine aminotransferase; ELISA, Enzyme-linked immunosorbent assay.

Metabolomic analysis identified L-Aspartic Acid as a candidate metabolite of interest. Therefore, hepatic L-Aspartic Acid concentration was further measured by ELISA. One-way ANOVA revealed a significant group difference in hepatic L-Aspartic Acid concentration (F _3, 26_ = 14.048, *P* < 0.01, η² = 0.618; **Figure 8A**). Compared with the CON group, the CUMS group showed a lower hepatic L-Aspartic Acid concentration (*P* < 0.01; **Figure 8A**). Compared with the CUMS group, TPM-like manipulations were associated with a higher hepatic L-Aspartic Acid concentration (*P* < 0.01; **Figure 8A**). FLX was also associated with a higher hepatic L-Aspartic Acid concentration (*P* < 0.05; **Figure 8A**).

To further examine the relationships among hepatic L-Aspartic Acid, liver-related indices, and depression-related behaviors, additional correlation analyses were performed. Pearson correlation analyses were then performed.

Specifically, hepatic L-Aspartic Acid concentration was positively correlated with plasma IGF-1 concentration (*R* = 0.5203, *P* < 0.01; **Figure 8B**). Moreover, hepatic L-Aspartic Acid concentration was positively correlated with SPP (*R* = 0.6212, *P* < 0.01; **Figure 8C**). Hepatic L-Aspartic Acid concentration was negatively correlated with plasma AST (*R* = -0.5556, *P* < 0.01; **Figure 8D**) and ALT (*R* = -0.5555, *P* < 0.01; **Figure 8E**). These findings suggest that higher hepatic L-Aspartic Acid levels were associated with higher plasma IGF-1 concentration, better behavioral performance, and lower liver injury-related indices.

## 4 Discussion

In the present study, CUMS induced depression in adolescent female rats, manifested as anhedonia-like and anxiety/depression-related behaviors, as well as impaired spatial learning and memory abilities. Moreover, CUMS-induced depression was also accompanied by the inflammation of the hippocampus and liver, disturbances in hepatic amino acid metabolism in adolescent female rats. In addition, CUMS was accompanied by inflammatory changes and reduced IGF-1 expression in the hippocampus, which is generally consistent with our previous studies (Wu et al., 2024; Zhang et al., 2025). TPM-like manipulations were associated with improved depressive-like behaviors, reduced inflammatory changes in the hippocampus and liver, and improved hepatic functional and metabolic indices. Meanwhile, TPM-like manipulations were associated with higher expression of IGF-1 and IGF-1R in the hippocampus, as well as higher hepatic GHR and IGF-1 expression. Importantly, the severity of depressive-like behaviors was correlated with hepatic L-Aspartic Acid levels and circulating IGF-1, supporting an association between liver metabolic alterations and behavioral outcomes, while not establishing causality.

An increasing number of studies have shown that the liver-brain axis plays an important role in the occurrence and development of depression. CUMS can induce liver inflammation and liver dysfunction (Xu et al., 2022; Pang et al., 2023). The pro-inflammatory cytokines induced by liver inflammation may cross the blood-brain barrier or stimulate nerves to contribute to neuroinflammation by activating central microglia (Takata et al., 2011; Pratim Das and Medhi, 2023). With the development of metabolomics technique in recent years, more and more studies have focused on the relationship between changes in liver metabolomics and depression. Liver metabolomics may capture subtle and early pathway-level alterations before overt histological or routine biochemical abnormalities appear, potentially making it sensitive to early liver injury and inflammation (Charkoftaki et al., 2022; Martínez-Sena et al., 2023). Previous clinical trials revealed that patients with depression demonstrated metabolomic abnormality in the circulation (Wang et al., 2024). Further animal experiment also revealed that CUMS induced metabolomic abnormality in the circulation and liver, which were closely related with depressive behaviors (Wu et al., 2016; Jia et al., 2023; Xie et al., 2023; Wang et al., 2024). The abnormality of amino acid metabolism is closely related with depression, since the disturbance of amino acid metabolism may contribute to the development of depression through inhibiting neurotransmitter synthesis and increasing neurotoxic metabolites (Parrott et al., 2016; Schopman et al., 2021). This study also found that CUMS was associated with alterations in amino acid-related metabolic pathways in early adolescent female rats, including aspartate, alanine and glutamate metabolism, cysteine and methionine metabolism, and aminoacyl-tRNA biosynthesis. Not all altered amino acids showed the same response pattern after TPM-like manipulations. L-Aspartic Acid, which was downregulated by CUMS, was significantly restored, whereas L-Methionine did not show significant recovery. This discrepancy is consistent with the essential amino acid status of L-Methionine: mammals cannot synthesize methionine *de novo* and instead rely on dietary intake together with the methionine cycle for its homeostasis (Sanderson et al., 2019; Lauinger and Kaiser, 2021). Although aspartate serves as a biosynthetic precursor for methionine in plants and microorganisms via the aspartate-derived pathway, this route is absent in mammalian systems (Jander and Joshi, 2009). In contrast, L-Aspartic Acid is a non-essential amino acid deeply integrated into endogenous metabolism, including tricarboxylic acid cycle anaplerosis and the malate-aspartate shuttle (Birsoy et al., 2015; Allen et al., 2016). Therefore, the selective restoration of L-Aspartic Acid but not L-Methionine suggests that TPM-like manipulations may modulate endogenous amino acid metabolism at specific metabolic nodes rather than broadly enhancing amino acid biosynthesis or altering exogenous amino acid absorption.

L-Aspartic Acid may also be relevant to liver protection because it is an important intermediate in the tricarboxylic acid and urea cycles and participates in energy metabolism and detoxification (Cappel et al., 2019; Nassef et al., 2021; Holeček, 2023; Su et al., 2024). Previous studies have suggested that L-Aspartic Acid may exert protective effects against liver injury by modulating inflammatory responses and inflammasome activity (Zhou et al., 2023). Additionally, L-Aspartic Acid has been reported to restore the activity of mitochondrial complexes I and II, enhance ATP production, and reduce the generation of reactive oxygen species in liver cells (Su et al., 2024).

In the present study, L-Aspartic Acid was identified as a candidate metabolite potentially associated with liver-brain communication; however, whether it acts as a mediator remains to be determined. The results of this study indicated CUMS significantly reduced hepatic L-Aspartic Acid levels. Moreover, hepatic L-Aspartic Acid was positively associated with IGF-1 levels in both the liver and circulation. Hepatic L-Aspartic Acid was also negatively associated with plasma AST and ALT levels, suggesting that reduced hepatic L-Aspartic Acid may be linked to liver injury under CUMS exposure and that L-Aspartic Acid may represent a protective metabolite in the liver. However, these correlations do not establish a direct causal role for L-Aspartic Acid in liver-brain communication. ALT and AST, as serum enzyme indicators, are sensitive markers of liver injury, primarily reflecting increased permeability of the hepatocyte membrane or cellular necrosis (Hu et al., 2022). Previous studies showed that CUMS caused the increased concentrations of ALT and AST in rats (Jia et al., 2016; Zhang et al., 2022a), which is consistent with our study. The gut-liver-brain axis may also be relevant (Sun et al., 2024). Current evidence suggests that short-chain fatty acids may confer hepatoprotective effects, primarily through anti-inflammatory actions, attenuation of hepatic lipid accumulation, improvement of mitochondrial function, and modulation of energy metabolism and metabolic balance (Li et al., 2024b). In our previous work, TPM-like manipulations may promote skeletal muscle growth in adolescent rats, potentially by modulating gut microbiota composition, increasing short-chain fatty acids production, and activating IGF-1-related signaling (Lin et al., 2024).

IGF-1 production peaks during adolescence and is important for somatic growth during this developmental period (Arroba et al., 2016). Brain IGF-1 is influenced in part by circulating IGF-1 (Torres-Aleman, 2010; Wan et al., 2022), which is produced predominantly by the liver (Puche and Castilla-Cortázar, 2012; Staerk et al., 2020). The binding of GH to GHR on the hepatocyte membrane triggers the gene transcription of IGF-1 in the liver (Fan et al., 2009; Hu et al., 2021). As a neurotrophic factor, IGF-1 also plays a crucial role in neurodevelopment, cognition, anti-neuroinflammation and anti-depression, which is mediated by its specific receptor IGF-1R (Li et al., 2019; Bhalla et al., 2022; Ge et al., 2022).

In the present study, TPM-like manipulations ameliorated depressive-like behaviors and was associated with improvement of hepatic amino acid metabolism, particularly with increased hepatic L-Aspartic Acid levels. The hippocampus is an important center for stress response and emotion regulation (Zhang et al., 2025). CUMS was accompanied by hippocampal inflammation and reduced expressions of IGF-1 and IGF-1R, consistent with previous studies (Ogundele et al., 2017; Li et al., 2024a; Zhang et al., 2025). Our previous study suggested that TPM-like manipulations were associated with antidepressant-like effects together with higher hippocampal IGF-1 expression and reduced neuroinflammatory changes. TPM-like manipulations were also associated with increased expressions of IGF-1 and IGF-1R in the hippocampus, together with increased hepatic and circulating IGF-1. These coordinated changes may be relevant to the beneficial behavioral and growth-related outcomes observed in CUMS-exposed early adolescent rats, although direct causal relationships were not established in the present study.

FLX, a selective serotonin reuptake inhibitor, is one of the most commonly used antidepressants in adolescents (Iñiguez et al., 2010). However, FLX may inhibit adolescent growth and development (Calarge et al., 2024) and cause liver damage (Soni and Mane, 2021). In this study, FLX still did not improve the growth of CUMS-exposed adolescent rats, although it improved depressive-like behaviors, which is also consistent with our previous study (Wu et al., 2024; Zhang et al., 2025). FLX was also associated with partial improvement in hepatic inflammatory changes and GHR/IGF-1-related indices, although residual inflammatory alterations and reduced GHR/IGF-1-related expression remained in the liver. In addition, FLX showed partial improvement in amino acid-related metabolic changes and was associated with a higher hepatic L-Aspartic Acid concentration. The absence of significant pathway enrichment in the FLX metabolomics data may suggest that its antidepressant effects are less prominently reflected in the present liver metabolomic profile and may involve mechanisms beyond the liver, including central pathways such as hippocampal inflammation-related processes. Therefore, the liver-related effects of FLX in this model should be interpreted cautiously and require further investigation.

There are several limitations to the present study. First, A major limitation of this study is that the proposed liver–brain axis mechanism remains correlational rather than causal. Without L-Aspartic Acid or IGF-1/IGF-1R intervention experiments, these factors should be considered candidate mediators rather than confirmed mechanisms. The regulatory role of L-Aspartic Acid in the liver–brain axis requires further validation. Secondly, this study did not measure plasma growth hormone or phosphorylated IGF-1R, limiting interpretation of upstream GH signaling and downstream IGF-1R activation. Third, the absence of a rigorously time-matched sham manipulation control means that non-specific handling effects cannot be fully excluded. Future studies should include a dedicated sham manipulation control to clarify the specific effects of spine-pinching manipulations. Exhaustion of the remaining liver tissue during revision also precluded additional immunohistochemical and immunofluorescence analyses for the further evaluation of hepatic inflammatory status. Fourth, the metabolomics analysis was exploratory, with a small sample size, limited supervised-model performance, and putative annotations based mainly on database matching. Finally, only female rats were studied. Because GH secretion, hepatic GHR/growth hormone-binding protein expression, and GH-regulated IGF-1 expression are sex-dependent in rats (Gabrielsson et al., 1995), the liver-related findings cannot be directly generalized to males. These sex-dependent differences may also affect the amino acid metabolism in the liver. Future studies should include male rats. Furthermore, although vehicle exposure was matched, 10% DMSO may have influenced hepatic readouts.

Taken together, the present study showed that CUMS-induced depressive-like behaviors were accompanied by the inflammation of hippocampus and liver, disturbances in hepatic amino acid metabolism, and alterations in GHR/IGF-1-related indices, which TPM-like manipulations prevented. However, due to limited sample size, the conclusion should be drawn cautiously and warrant further study. The observed correlations among hepatic L-Aspartic Acid, IGF-1-related indices, liver injury markers, and behavioral outcomes suggest a potential involvement of the liver-brain axis in the effects of TPM-like manipulations.

## 5 Conclusion

In this study, TPM-like manipulations prevented depressive-like behaviors, liver and hippocampal inflammatory changes, abnormal hepatic amino acid metabolism as well as the reduction in hepatic GHR/IGF-1-related expression in CUMS-exposed adolescent female rats. Moreover, the results in this study also suggest that the regulation of liver-brain axis may be involved in the antidepressant effects of TPM-like manipulations, where L-Aspartic Acid and IGF-1 may be candidate molecules. However, due to limited sample size, a confirmative conclusion still warrants a further study.

## Data Availability

The data presented in the study are deposited in the MetaboLights repository, accession number MTBLS13600.
