# Supplementary material for: TPM-like manipulations exerted an antidepressant effect and regulated amino acid metabolism and GHR/IGF-1 pathway in the liver in adolescent female CUMS rats
Source: Front Physiol. 2026 May 14;17:1752608. doi: 10.3389/fphys.2026.1752608 (PMC13215830; doi:10.3389/fphys.2026.1752608)
Supplement: Supplementary file 1 [file DataSheet1.pdf]

Supplementary Material

Supplementary Figure 1

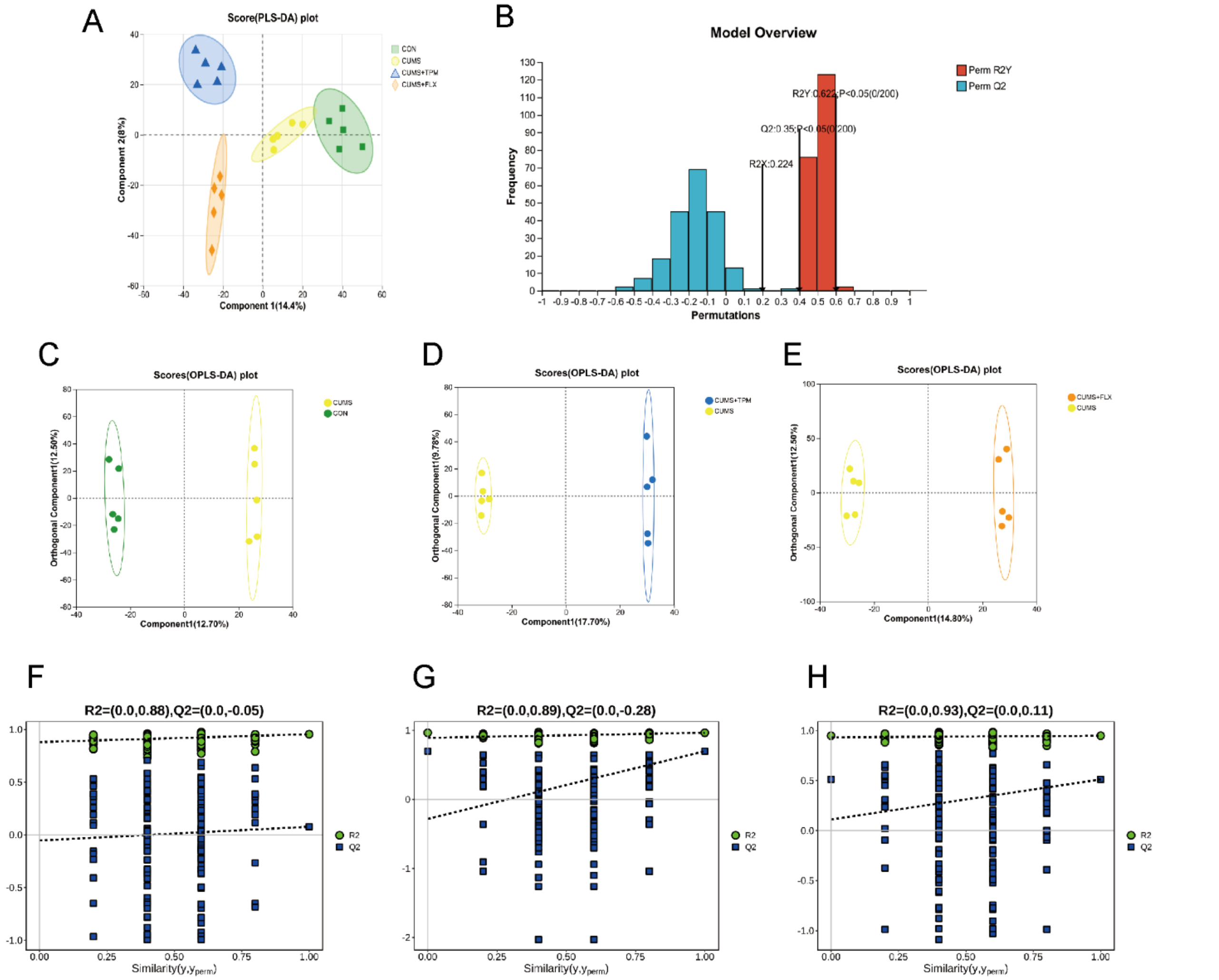

Supplementary Figure 1. (A) Plots of PLS-DA score for the CON, CUMS, CUMS + TPM, and CUMS + FLX groups; (B) Plots of PLS-DA replacement tests for the CON, CUMS, CUMS + TPM, and CUMS + FLX groups; (C) Plots of OPLS-DA score for the CON and CUMS groups; (D) Plots of OPLS-DA score for the CUMS and CUMS + TPM groups; (E) Plots of OPLS-DA score for the CUMS and CUMS + FLX groups (n = 5); (F) Permutation test results of the OPLS-DA model for the CON group versus the CUMS group. (G) Permutation test results of the OPLS-DA model for the CON group versus the CUMS + TPM group. (H) Permutation test results of the OPLS-DA model for the CON group versus the CUMS + FLX group.Abbreviations: CON, control; CUMS, chronic unpredictable mild stress; TPM, traditional pediatric massage-like manipulations; FLX, fluoxetine.Permutation test results of the OPLS-DA model for liver metabolomics.
